# A new QEXAFS system on the general XAFS beamline at the Shanghai Synchrotron Radiation Facility

**DOI:** 10.1107/S1600577522008177

**Published:** 2022-10-10

**Authors:** Yongnian Zhou, Songqi Gu, Ying Zhao, Zheng Jiang, Zhaohong Zhang

**Affiliations:** a Shanghai Advanced Research Institute, Chinese Academy of Sciences, Shanghai 201210, People’s Republic of China; University of Essex, United Kingdom

**Keywords:** SSRF, XAFS, QEXAFS, *EPICS*

## Abstract

A new quick-scanning extended X-ray absorption fine-structure (QEXAFS) system for *in situ* studies has been developed and tested on the general XAFS beamline at the Shanghai Synchrotron Radiation Facility. In the new system, an analog-to-digital converter (ADC) with 1 MHz sampling rate is used to acquire the detector data while one scaler is used to precisely calculate the scanning energy. Two external hardware trigger signals were adopted to synchronize the data collection of the ADC and the scaler. The software development platforms of the double-crystal monochromator control system and the new QEXAFS system have been unified with the Experimental Physics and Industrial Control System. By comparing the spectra acquired by the conventional step-by-step XAFS system with an energy range of 1200 eV at the 7.5um Cu foil *K*-edge, the new system demonstrates satisfactory signal-to-noise ratio and energy resolution. The previous shortcomings, including distortion of the spectrum and energy shift, have been overcome. The tests with different integration times indicated that appropriate parameters not only ensure good experimental results but also enhance the smoothness of the XAFS spectrum at high energy zones. The reliability of the new system has also been verified.

## Abstract 2

A new quick-scanning extended X-ray absorption fine-structure (QEXAFS) system for *in situ* studies has been developed and tested on the general XAFS beamline at the Shanghai Synchrotron Radiation Facility. Tests with different integration times indicated that appropriate parameters not only ensure good experimental results but also enhance the smoothness of the XAFS spectrum at high-energy zones.

## 1. Introduction

Quick-scanning extended X-ray absorption fine structure (QEXAFS) (Frahm, 1988 ▸, 1989 ▸) is a powerful structural analysis tool for time-resolved *in situ* investigations of materials on the atomic scale during physical and chemical reaction processes (Grandjean *et al.*, 2005 ▸; Stötzel *et al.*, 2011 ▸; Pao *et al.*, 2021 ▸). Its inherent advantages, including short time scanning periods, low radiation dose, information on kinetics, and the elucidation of chemical-reaction and phase-transition mechanisms, allow QEXAFS to play an important role in the field of time-resolved studies (Eslava *et al.*, 2016 ▸; Prestipino *et al.*, 2011 ▸; Gaur *et al.*, 2019 ▸; Marchionni *et al.*, 2020 ▸). Differing from the sophisticated setups of energy-dispersive XAFS (Flank *et al.*, 1982 ▸) and pump–probe XAFS (Oyanagi *et al.*, 2001 ▸; Bressler *et al.*, 2009 ▸; Uemura *et al.*, 2020 ▸; Smolentsev *et al.*, 2018 ▸), the QEXAFS method can be developed on the general XAFS beamline and shares most of the devices with the conventional XAFS experimental method.

As a time-resolved method, QEXAFS has been considerably evaluated and realized at the XAFS beamline. The first QEXAFS system was based on *LabVIEW* (Zou *et al.*, 2009 ▸). Using the system, an EXAFS spectrum of a Cu metal foil at the Cu *K*-edge can be acquired in less than 5 s, which shows a great improvement in the experimental efficiency time resolution of the XAFS beamline. Because every energy datapoint of the XAFS spectrum is calculated according to the parameters of the time interval and the moving speed of the Bragg motor, it cannot represent the real energy during the experiment. In order to overcome this issue, we adopted the IK220 card to obtain each energy datapoint during the movement of the Bragg motor. In this improvement, the data acquisition system (Liu *et al.*, 2012*a* ▸,*b* ▸) was still based on *LabVIEW*.

However, the beamline control system at the Shanghai Synchrotron Radiation Facility (SSRF) is developed with *EPICS* which is very popular in synchrotron radiation facilities all over the world. So there exists a time difference between the experimental data acquired with *LabVIEW* and the corresponding X-ray energy controlled by *EPICS*. In this new QEXAFS system, we integrated the data acquisition part with *EPICS* (Zhou *et al.*, 2014 ▸, 2015 ▸). Furthermore, the external hardware trigger signals with 1 MHz are used to synchronize precisely for acquiring the detector data with the scanning energy. A graphical user interface (GUI) is provided for the users to complete the experiment. The test results show that the improvements of software and hardware not only ensure a satisfactory signal-to-noise ratio for the EXAFS spectrum but also enhance the stability of the system.

## 2. Wiggler source of the XAFS beamline

The XAFS beamline is one of the first seven beamline stations built by SSRF. It is a general and high-performance X-ray absorption beamline station based on a multipole wiggler light source and is mainly used for conventional X-ray fine structure spectroscopy research, taking into account the combined research methods of XAFS, X-ray diffraction (XRD) and X-ray scattering. The main performance specifications of the XAFS beamline are shown in Table 1 ▸ (Yu *et al.*, 2015 ▸).

**Figure 1:**
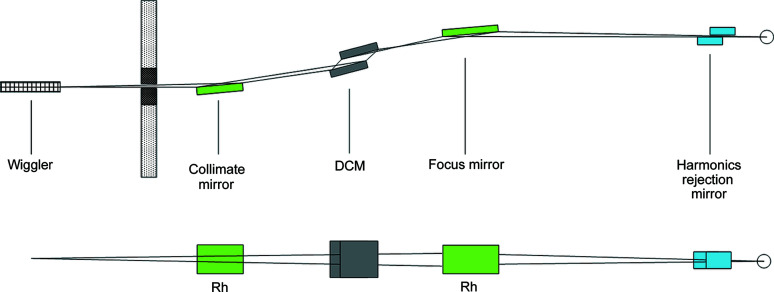
Schematic of the XAFS beamline in focused mode.

The XAFS beamline has two operation modes, namely the focused mode and the unfocused mode. In the focused mode, the main optical elements include a collimating mirror, a liquid nitro­gen-cooled double-crystal monochromator (DCM), a focusing mirror and a harmonics rejection mirror. The layout of the XAFS beamline in focused mode is shown schematically in Fig. 1 ▸.

## 3. Design of the hardware system

### 3.1. Architecture

**Figure 2:**
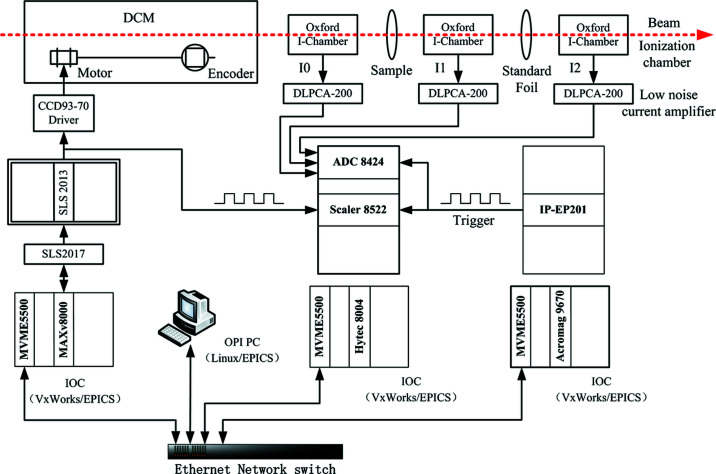
Schematic overview of the QEXAFS system (right-hand part) and the control system of the Bragg motor (left-hand part).

Fig. 2 ▸ shows a schematic of the QEXAFS system (the right-hand part) and the control system of the Bragg motor (the left-hand part). The input and output controller (IOC) of the control system is composed of a VME crate, a MVME 5500 single-board computer and an eight-axis motor controller MAXv-8000. It runs the VxWorks real-time operation system (RTOS) and the *EPICS* distributed control system (DCS). The control pulses of the MAXv-8000 are transferred via the step motor driver interface (SLS 2017 and SLS 2013) and divided into two parts: one is for the CCD93-70 step motor driver to control the Bragg motor, the other is used for the input signal of the scaler.

The QEXAFS system adopts two IOCs: one is for the analog-to-digital converter (ADC) and the scaler, the other is for the external hardware signal source. The ADC (Hytec 8424) and the scaler (Hytec 8522) are both industry pack (IP) cards, and their carrier boards are Hytec 8004. The ADC card provides four channels of simultaneously sampled analogue digitalization with up to 1 MHz sampling rate. It is used to acquire the current signals from three ionization chambers. The scaler card is used to record the number of control pulses during the movement of the Bragg motor and provide its relative distance and energy offset for the XAFS spectrum. The other IOC is used for the Acromag AVME9670 carrier board and IP-EP201 reconfigurable digital IP card which provides the external trigger signals to the ADC and the scaler.

### 3.2. ADC

The ADC plays an important role in the QEXAFS system, having a large influence on performance. Table 2 ▸ shows its main characteristics. According to our previous work, the Hytec 8424 ADC card can fully meet the requirements of our QEXAFS system.

The beam current signals include *I*_0_ (before the sample), *I*_1_ (after the sample) and *I*_2_ (after the standard foil). The absorption coefficient of the sample is calculated according to the following equation,

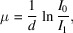

where *d* is the thickness of the sample.

When working under 1 MHz sampling rate the raw data acquired by the ADC are numerous. This will lead to great difficulty in data transmission, processing and storage. The *EPICS* driver program carried out the compression by averaging the data over a defined period of time in order to reduce the size of the experimental data and improve the efficiency of the system.

### 3.3. Scaler

The scaler card is a single-width IP card that provides 16 independent counting channels with up to 100 MHz for TTL input signals. It can be used to count the control pulses from the MAXv-8000 controller and calculate the relative distance of the Bragg motor and the energy point corresponding to each absorption coefficient. Like the ADC, there is also the average function in the *EPICS* driver program. The mean value is a representation of the accumulated value during a defined period of time.

**Figure 3:**
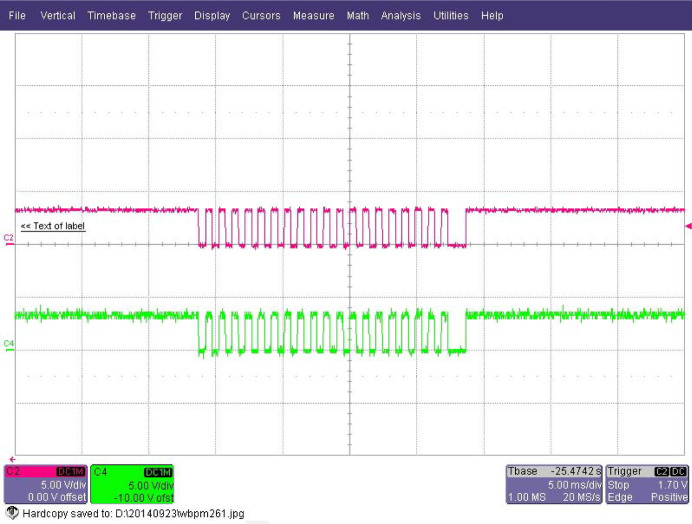
Two channels of control pulse signal output from the SLS2013: one is for the CCD93-70 step motor driver to control the Bragg motor of the DCM, the other is for the input signal of the scaler.

In the laboratory we set up the same architecture to test the scaler with a step motor stage. The control pulses from the MAXv-8000 were divided into two parts, one was input to the step motor stage and the other was input to the scaler. The stage moves 0.5 µm per control pulse and is set to move 10 µm. Fig. 3 ▸ shows the measurement results of the oscilloscope. We can see there are 20 control pulses detected which is exactly consistent with the count result of the scaler.

### 3.4. Trigger signal source

**Figure 4:**
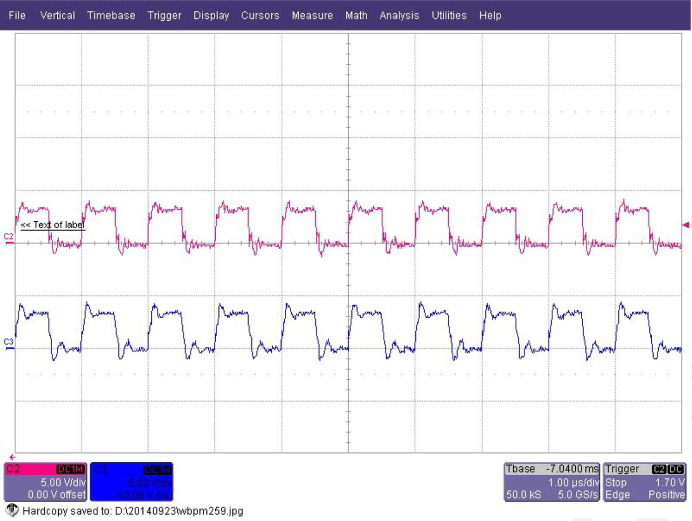
Two channels of hardware trigger signals: one is for the ADC, the other is for the scaler.

The external hardware trigger signal source is used to provide the synchronous external trigger signals for the ADC and the scaler. The frequency of the trigger signals is set to 1 MHz according to the ADC sampling rate. This ensures a one-to-one complete coincidence for each result of the ADC and the scaler. Fig. 4 ▸ shows two hardware trigger signals.

## 4. Design of the software system

### 4.1. Flow pattern

**Figure 5:**
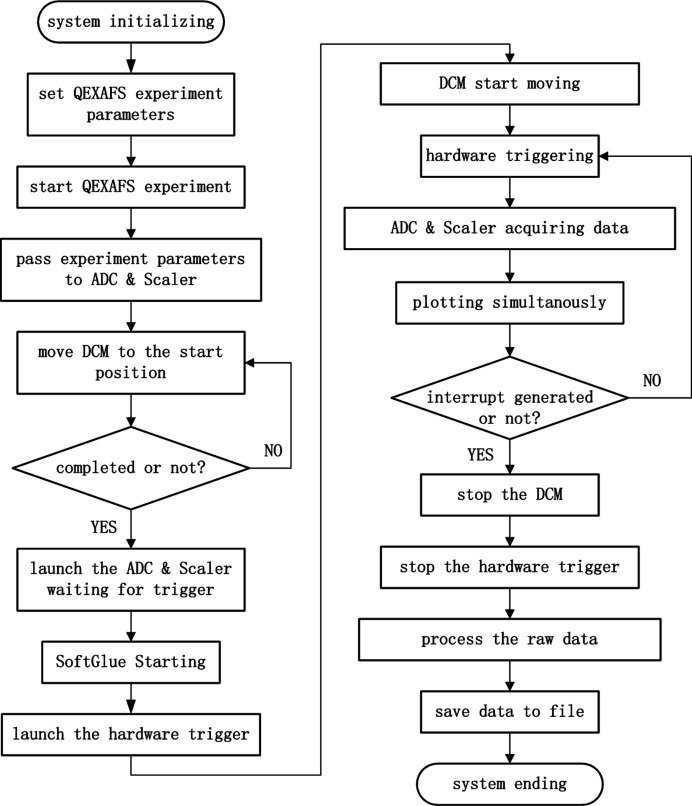
Software flow pattern of the QEXAFS system.

A QEXAFS experimental procedure includes the initialization of the hardware system, setting appropriate parameters, energy scanning, acquiring detector data and so on. These functions are realized using Python, a popular and powerful programming language. Fig. 5 ▸ shows the flow pattern of the QEXAFS system.

### 4.2. Main GUI

**Figure 6:**
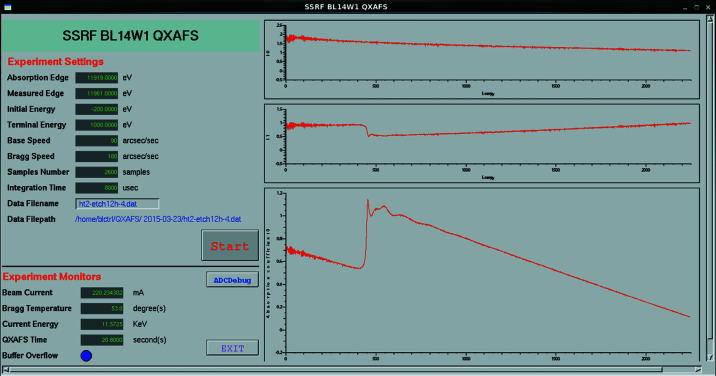
Main GUI of the QEXAFS system.

Fig. 6 ▸ shows the main GUI of the QEXAFS system, designed using the *Extensible Display Manager* (*EDM*; Sinclair, 2007 ▸) which is a frequently used tool in *EPICS*. The GUI has three main components: the setting section for experimental parameters, the monitoring section for the experimental environment, and the displaying section for the results. The setting section is located in the upper left and defines all of the experimental parameters including the absorption edge of the sample, the calibration parameters, the moving speed of the Bragg motor, the sampling number, the integration time, and the datafile name and location. The sampling number parameter determines the number of saved data. The integration time determines the time period for which each dataset is averaged. The monitoring section is under the setting section and provides information about the beam current, temperature of the Bragg motor, present energy, absorption time and buffer status. The displaying section is on the right of the main GUI and is used to display the experimental results including *I*_0_ (before the sample), *I*_1_ (after the sample) and the absorption coefficient (calculated from *I*_0_ and *I*_1_). The experimental results shown here are generally used for qualitative observation for users. If the experimental data are needed for quantitative study, it is necessary to use XAFS spectrum analysis software, such as *IFEFFIT* (Newville, 2001 ▸; Ravel & Newville, 2005 ▸).

## 5. Results and discussion

To illustrate the performance of the QEXAFS system, we performed three kinds of experiments with a standard copper foil at the Cu *K*-edge: (*a*) comparison between the QEXAFS system and the conventional XAFS system; (*b*) comparison of different integration times with the QEXAFS system; (*c*) comparison of the QEXAFS system tested five times. The experimental conditions are as shown in Table 3 ▸.

According to Bragg’s law (Batterman & Cole, 1964 ▸), the movement of the DCM is 5716 arcsec and takes less than 7.94 s.

### 5.1. Comparison of QEXAFS with conventional XAFS

**Figure 7:**
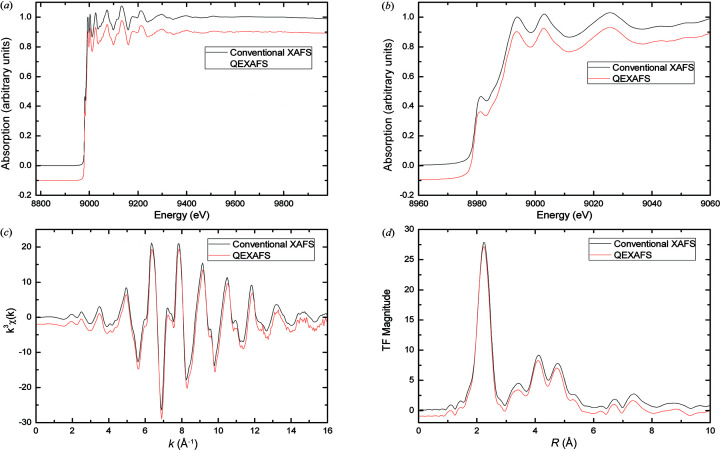
Comparison of the Cu *K*-edge XAFS spectra with a 1200 eV energy range measured with the QEXAFS system in less than 8 s and the conventional XAFS system in nearly 30 min. (*a*) Normalized XAFS spectra, (*b*) near-edge part, (*c*) *k*-space spectra, (*d*) *R*-space spectra.

Fig. 7 ▸ shows the XAFS spectra of the Cu *K*-edge with an energy range of 1200 eV obtained by the QEXAFS system and the conventional XAFS system. Fig. 7 ▸(*a*) indicates that the QEXAFS spectrum has the same waveform, including absorption peaks and oscillations, as the conventional XAFS spectrum. Fig. 7 ▸(*b*) illustrates the near-edge part of the QEXAFS spectrum and the conventional XAFS spectrum. The clear shoulder peak of the QEXAFS spectrum shows that the energy resolution of the QEXAFS system is good enough compared with the conventional XAFS system. Fig. 7 ▸(*c*) shows the *k*-space spectra of the QEXAFS spectrum and the conventional XAFS spectrum, where *k* is the wavevector of the ejected photoelectron. The curve of the *k*-space spectrum demonstrates that the QEXAFS spectrum has a clear periodicity and a good smooth performance when *k* = 16. After completing Fourier transformation, the *k*-space spectra are transformed to *R*-space spectra, as shown in Fig. 7 ▸(*d*). The same intensities and locations of their coordination peaks with the two methods shows that the experimental results of the QEXAFS system are reliable and persuasive.

### 5.2. Effect of integration time

**Figure 8:**
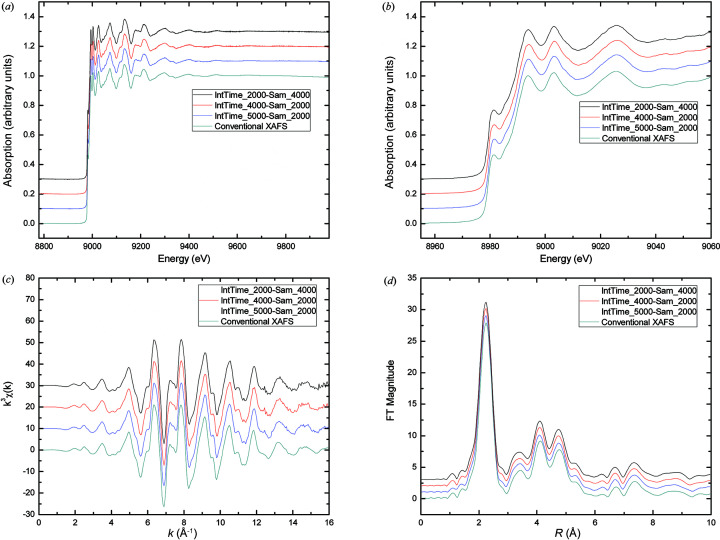
Comparison of the Cu *K*-edge XAFS spectra with a 1200 eV energy range measured by the QEXAFS system with different integration times and the conventional XAFS system. (*a*) Normalized XAFS spectra, (*b*) near-edge part, (*c*) *k*-space spectra, (*d*) *R*-space spectra.

The parameter of integration time has different meanings to the ADC and the scaler. For the ADC, the integration time is the averaged photoelectron current over a period of time, whereas for the scaler it signifies the accumulated number of input pulses corresponding to a certain number of triggers. Noise, as a random interference signal which is not expected in the QEXAFS system, can be decreased to an extent when the integration time is sufficiently improved. However, excessive integration time will lead to loss of high-frequency components of the signal. We obtained three QEXAFS spectra in integration times of 2000 µs, 4000 µs and 5000 µs. These QEXAFS spectra, together with the conventional XAFS spectrum, are shown in Fig. 8 ▸(*a*), and their near-edge parts are shown in Fig. 8 ▸(*b*). The results indicate that they all have the same shape waveform. Furthermore, the shoulder peaks of the QEXAFS spectra are all obvious.

Figs. 8 ▸(*c*) and 8 ▸(*d*) show the *k*-space results and *R*-space results, respectively. The results in Fig. 7 ▸(*c*) demonstrate that all of the QEXAFS spectra have a good smooth performance when *k* = 16. When the integration time is set to 5000 µs, the smooth performance is also enhanced. The same locations of coordination peaks, as shown in Fig. 7 ▸(*d*), prove that the different integration times have no obvious effect on the *R*-space spectrum.

### 5.3. Reliability of the QEXAFS system

**Figure 9:**
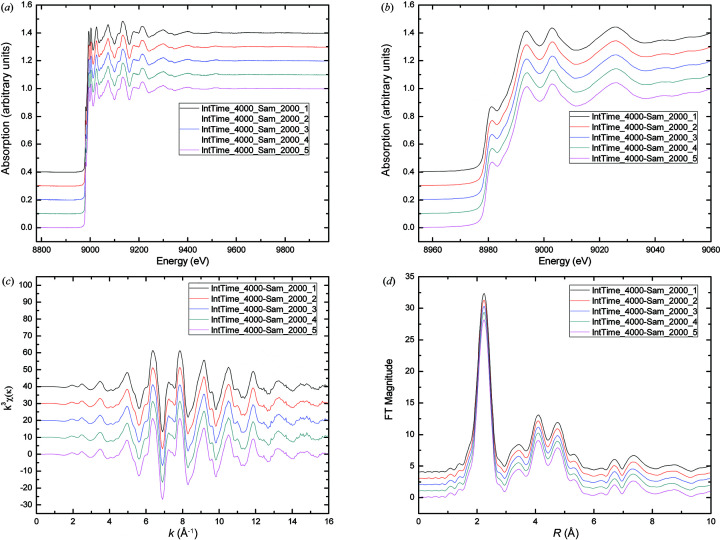
Cu *K*-edge XAFS spectra with a 1200 eV energy range measured by the QEXAFS system under the same conditions. (*a*) Normalized XAFS spectra, (*b*) near-edge part, (*c*) *k*-space spectra, (*d*) *R*-space spectra.

The repeatability of the spectra under the same experimental conditions is very important for *in situ* studies and is largely determined by the stability of the data acquisition system. We carried out the experiments five times under the same conditions with the standard 7.5 µm copper foil. The results shown in Fig. 9 ▸ indicate that the QEXAFS system has very good stability. The experimental results, which are illustrated using normalized spectra, or the spectra in *k*-space and the spectra in *R*-space, reveal a very good repeatability under the same experimental conditions.

## 6. Conclusions

A new QEXAFS system on the general XAFS beamline at the SSRF has been developed. The ADC is used to obtain the photoelectron current signals before and after the sample while the scaler is used for counting the number of the control pulses corresponding to the movements of the Bragg motor. They work synchronously with two trigger signals which are provided by one FPGA-based module. The software of the new QEXAFS system is developed with *EPICS* which is adopted in the beamline control system of SSRF. By comparing with the spectra acquired using the conventional XAFS system, we demonstrated that the new QEXAFS system maintains a satisfactory signal-to-noise ratio but improves the capability of the energy resolution. The experimental results with different integration times show that setting this parameter appropriately can improve the quality of the spectrum. The excellent stability of the system and the fine repeatability of the spectra are also proved. The successful implementation of the new QEXAFS system has a great practical significance for carrying out fast time-resolved *in situ* studies at SSRF.

## Supplementary Material

Raw QEXAFS data. DOI: 10.1107/S1600577522008177/rv5164sup1.bin

## Figures and Tables

**Table 1:** Main performance specifications of the XAFS beamline

Equipment	Performance specifications
Light source	38-pole wiggler
Electron energy	3.5 GeV
Magnetic field intensity	1.2 T
Beam intensity	250 mA
Beamline acceptance angle	1.0 mrad × 0.1 mrad (H × V)
Energy range	4.5–20 keV, Si(111), focused; 8–40 keV, Si(311), unfocused
Energy resolution (Δ*E*/*E*)	1.5 × 10^−4^ Si(111) at 10 keV
Flux at sample	5.0 × 10^12^ at 10 keV, Si(111), focused, 250 mA
Focused spot size	0.3 mm × 0.3 mm (H × V)
High harmonic content	10^−4^ harmonic suppression mirror (focused)
Detection mode	Transmission, fluorescence

**Table 2:** Main characteristics of the ADC

Index	Characteristic
1	Four independent channels
2	Up to 1 MHz sampling rate
3	Resolution: 16 bits no missing codes
4	Accuracy: 14 bits
5	On-board RAM memory 128 K × 32bits × 4 channels
6	Programmable full-scale resolution all inputs ±10 V or ±5 V

**Table 3:** Experimental conditions used in this work

Parameter	Experimental condition
Energy of the storage ring	3.5 GeV
Beam current	200 mA
Flux (photons s^−1^)	10^12^ photons s^−1^ (0.1% bandwidth)^−1^
Sample	7.5 µm standard copper foil
DCM crystal type	Si(111)
Speed of the Bragg motor	720 arcsec s^−1^
Initial energy	8779 eV (*E*_k_ − 200 eV)
Terminal energy	9979 eV (*E*_k_ + 1000 eV)
